# Supplementary material for: Efficacy of Topical Essential Oils in Musculoskeletal Disorders: Systematic Review and Meta-Analysis of Randomized Controlled Trials
Source: Pharmaceuticals (Basel). 2023 Jan 19;16(2):144. doi: 10.3390/ph16020144 (PMC9959659; doi:10.3390/ph16020144)
Supplement: Supplementary file 1 [file pharmaceuticals-16-00144-s001.zip › Figure S1.pdf]

Studies with intention-to-treat

Clinical trial	Randomization process	Deviations from intended interventions	Missing outcome data	Measurement of the outcome	Selection of the reported result	Overall
Eftekharsadat, 2017	+	?	+	–	+	–
Nasiri, 2016	+	?	+	–	+	–
Kong, 2012	+	?	+	+	?	!
Shirazi, 2017	+	?	+	–	+	–
Yip, 2008	+	?	+	+	?	!

+ Low risk

? Some concerns

– High risk

A

Studies with pre-protocol

Clinical trial	Randomization process	Deviations from intended	Missing outcome data	Measurement of the outc	Selection of the reported	Overall
Ou, 2014	+	+	+	–	?	–
Pehlivan, 2019	+	–	+	–	?	–

+ Low risk

? Some concerns

– High risk

B
