# Supplementary material for: Efficacy of Topical Essential Oils in Musculoskeletal Disorders: Systematic Review and Meta-Analysis of Randomized Controlled Trials
Source: Pharmaceuticals (Basel). 2023 Jan 19;16(2):144. doi: 10.3390/ph16020144 (PMC9959659; doi:10.3390/ph16020144)
Supplement: Supplementary file 1 [file pharmaceuticals-16-00144-s001.zip › Figure S2.pdf]

Studies with intention-to-treat

Unique ID	Randomization process	Deviations from intended interventions	Missing outcome data	Measurement of the outcome	Selection of the reported result	Overall
Pehlivan	+	−	+	−	?	−
Yip 2008	+	?	+	+	?	!
Yip 2006	+	−	+	−	?	−

+ Low risk

? Some concerns

− High risk
