# Supplementary material for: Efficacy of Topical Essential Oils in Musculoskeletal Disorders: Systematic Review and Meta-Analysis of Randomized Controlled Trials
Source: Pharmaceuticals (Basel). 2023 Jan 19;16(2):144. doi: 10.3390/ph16020144 (PMC9959659; doi:10.3390/ph16020144)
Supplement: Supplementary file 1 [file pharmaceuticals-16-00144-s001.zip › Table S2.pdf]

Certainty assessment							Summary of findings				
**Participants (studies)**	**Risk of bias**	**Inconsistency**	**Indirectness**	**Imprecision**	**Publication bias**	**Overall certainty of evidence**	**Study event rates (%)**		**Relative effect (95% CI)**	**Anticipated absolute effects**	
**Follow-up**							**With placebo**	**With essential oil therapy**		**Risk with placebo**	**Risk difference with essential oil therapy**
**Pain intensity (assessed with: VAS; Scale from: 0 to 10)**											
443 (7 RCTs)	serious[a]	very serious[a]	not serious	not serious	none	⨁◯◯◯ Very low	222	221	-	The mean pain intensity was **-0.64**	MD **0.64 lower** (0.99 lower to 0.3 lower)
**Stiffness (assessed with: VAS; Scale from: 0 to 8)**											
124 (3 RCTs)	serious[a]	very serious[a]	not serious	serious[a]	none	⨁◯◯◯ Very low	64	60	-	The mean stiffness was **-0.77**	MD **0.77 lower** (1.57 lower to 0.04 higher)
