# Supplementary figures and images for: Efficacy of Topical Essential Oils in Musculoskeletal Disorders: Systematic Review and Meta-Analysis of Randomized Controlled Trials
Source: Pharmaceuticals (Basel). 2023 Jan 19;16(2):144. doi: 10.3390/ph16020144 (PMC9959659; doi:10.3390/ph16020144)

## Slide 1
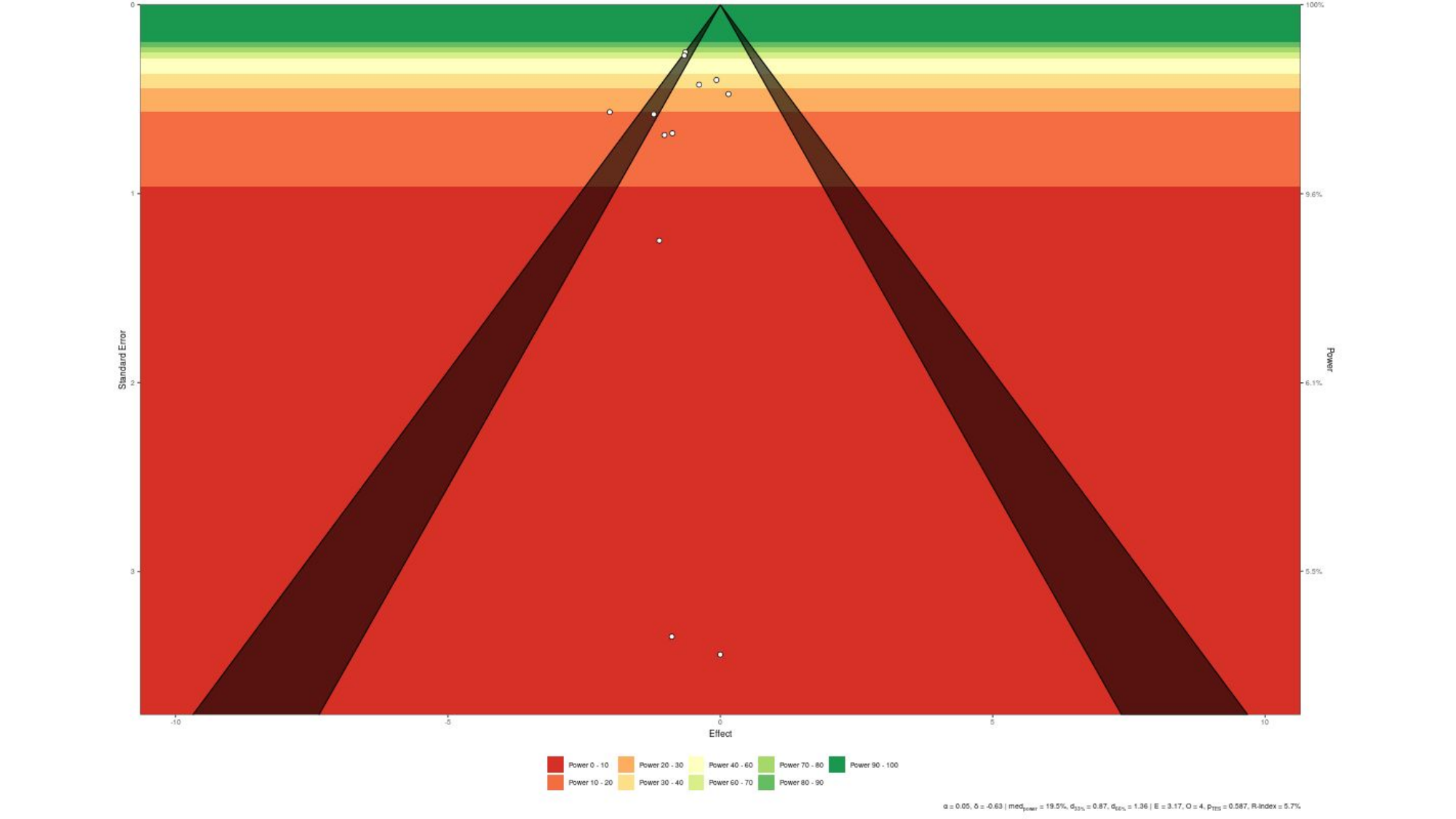

Supplement: Supplementary file 1 [file pharmaceuticals-16-00144-s001.zip › Figure S3.pptx]
